# Successful Management of Chronic Chylothorax Secondary to Gorham-Stout Disease

**DOI:** 10.1016/j.atssr.2024.06.020

**Published:** 2024-07-04

**Authors:** Liam D. Hyland, Abdelrahman Elsayed, Mohammad Hawari

**Affiliations:** 1 Thoracic Surgery Department, Nottingham University Hospitals NHS Trust, UK

## Abstract

Gorham-Scout disease (GSD) is a rare skeletal disorder of unknown etiology characterized by progressive osteolysis and excessive lymphovascular proliferation. Chylothorax is a life-threatening complication. A teenager presented with a left pleural effusion on a background of chronic flank collection secondary to lymphovascular malformation. Cytology of the fluid confirmed a chylothorax and rib excision biopsy confirmed GSD. The patient underwent thoracotomy, decortication, and pleurectomy, which helped to stabilize the chylothorax; ongoing management involved radiotherapy and bisphosphonates. GSD is a rare disease that can develop a number of severe complications including chylothorax. Management via surgical and radiotherapeutic techniques can provide long-term remission.

Gorham-Stout disease (GSD) is a rare skeletal disorder of unknown etiology characterised by progressive osteolysis and excessive lymphovascular proliferation.^1^ Its bony destructive process was first described as a concept in 1838 and subsequently classified as an entity in 1955.^2^ Chylothorax is a severe and life-threatening complication of GSD that confers a poor prognosis. We report the case of a teenager who presented with a large pleural effusion, confirmed to be a chylothorax secondary to GSD. A number of surgical and oncologic interventions were performed that successfully stabilized the chylothorax and improved the patient’s clinical status.

A 16-year-old female patient initially presented to the emergency department with increased dyspnea and chest pain. This was on a background of previous large left pleural effusion and longstanding left flank collection associated with a lymphangiomatous malformation since the age of 5 years. The patient had previously undergone spinal rod fixation surgery for eroded and collapsed vertebrae 2 years prior in another center outside of the United Kingdom; she also underwent drainage of the left flank collection and large left pleural effusion 6 months before. There were no specific details recorded on the indications nor outcomes from these procedures.

There was no history of tuberculosis, nor did she smoke. Clinical examination revealed significantly reduced air entry on the left side and a fluctuant swelling palpable in the left flank. Chest radiograph showed a large left pleural effusion with some collapse of the underlying lung (Figure 1A). Computed tomography of thorax/abdomen revealed a large recurrent left pleural effusion with tracheal deviation and mediastinal shift (Figure 1B); lytic lesions were also seen throughout T7-T12 vertebrae and left-sided posterior ribs (Figure 1C). Initial clinical suspicion was that of tuberculosis, atypical infection or lymphangiomatous lesion. Diagnostic aspiration and subsequent cytologic analysis confirmed that the fluid was consistent with that of a chylothorax.

**Figure 1:**
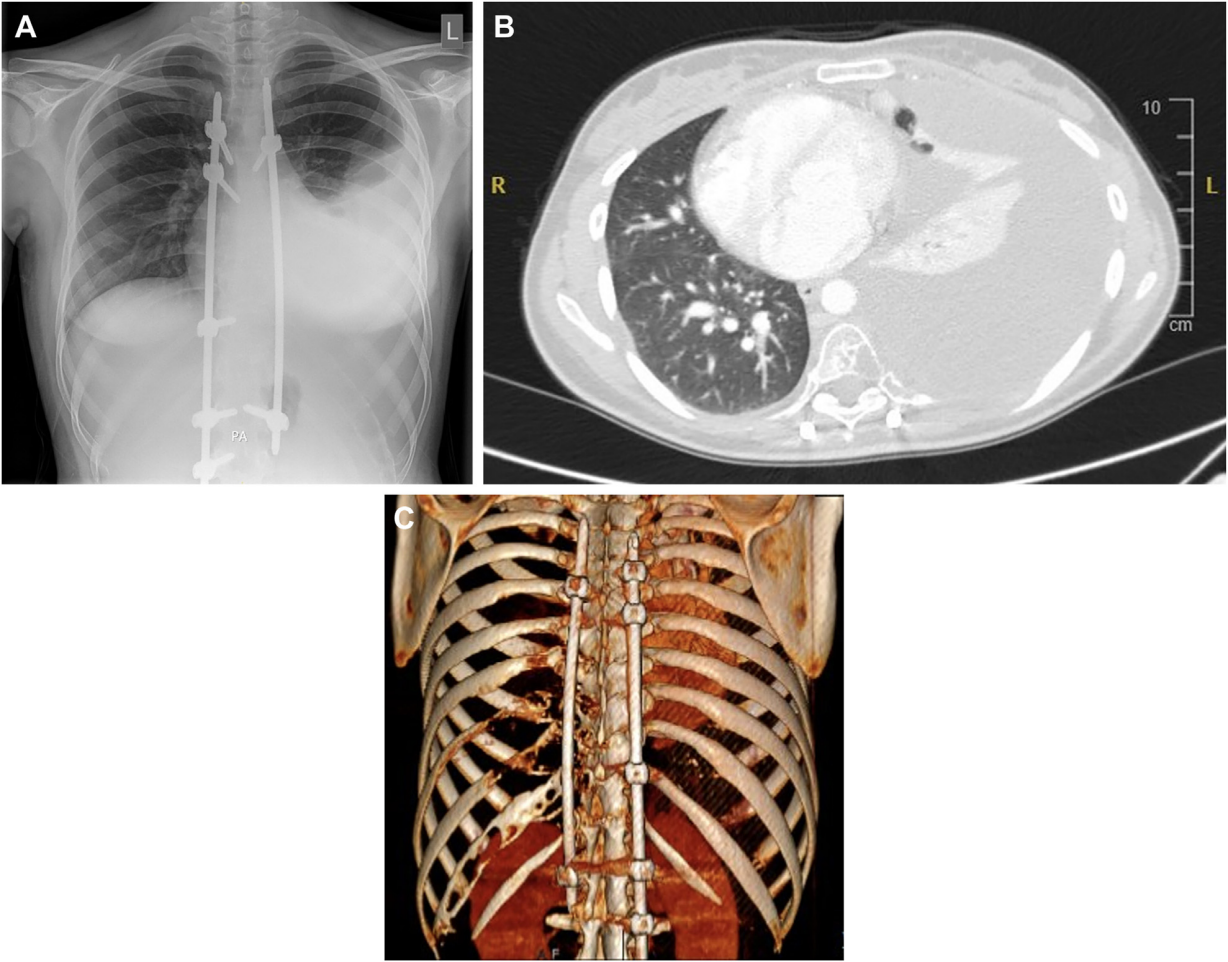
(A) Posteroanterior chest radiograph showing large left-sided pleural effusion as well as spinal rods and screws from previous surgery. (B) Computed tomography of the thorax demonstrating mediastinal shift due to the large left-sided pleural effusion. (C) Three-dimensional reconstruction showing lytic lesions dispersed throughout T7-T12 vertebrae and left ribs 8-11.

The patient subsequently underwent left video-assisted thorascopic surgery drainage of the chylothorax alongside multiple pleural biopsies. Intraoperatively, widespread osteolytic lesions were seen throughout the posterior ribs and the effusion was seen to be communicating with the collection situated in the left flank. Postoperatively, the patient drained approximately 1 L of chyle and was placed on a low-fat diet. Repeat computed tomography of the thorax showed dramatic improvement of the left pleural effusion with the chest drain in situ (Figure 2). The drain was eventually removed 3 weeks later and, following this, there was an interval increase in the size of the left flank mass.

**Figure 2:**
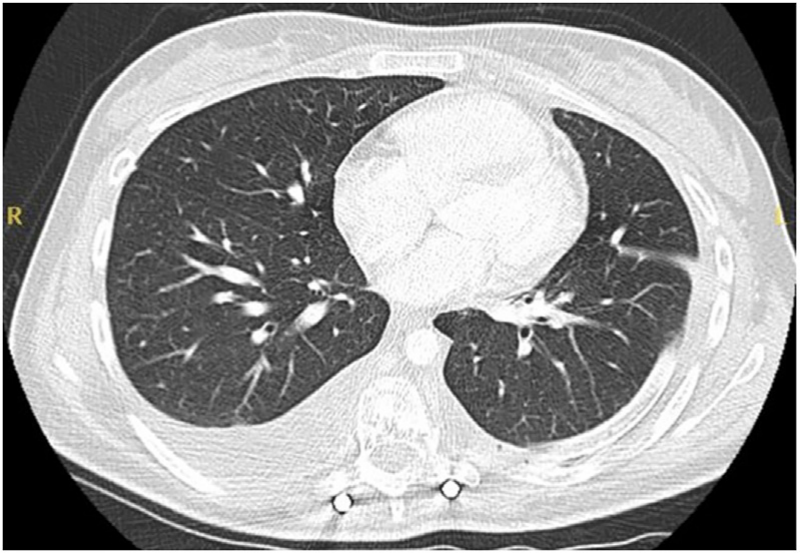
Computed tomography of the thorax demonstrating resolution of the left pleural effusion with outline of chest drain in situ.

Other surgical management options were deliberated and, 3 months later, the patient was deemed to be a suitable candidate for thoracic duct ligation and chest drain insertion. Thoracic duct ligation was carried out via a right video-assisted thorascopic surgery approach followed by talc pleurodesis 1 week later. An excision biopsy of a left posterior rib (Figure 3A) was carried out a few weeks later which helped to confirm the diagnosis of GSD based upon histologic analysis. The patient subsequently underwent a course of radiotherapy 1 month later at which she received 40 Gy in 20 fractions to the thoracic spine and left chest wall.

**Figure 3:**
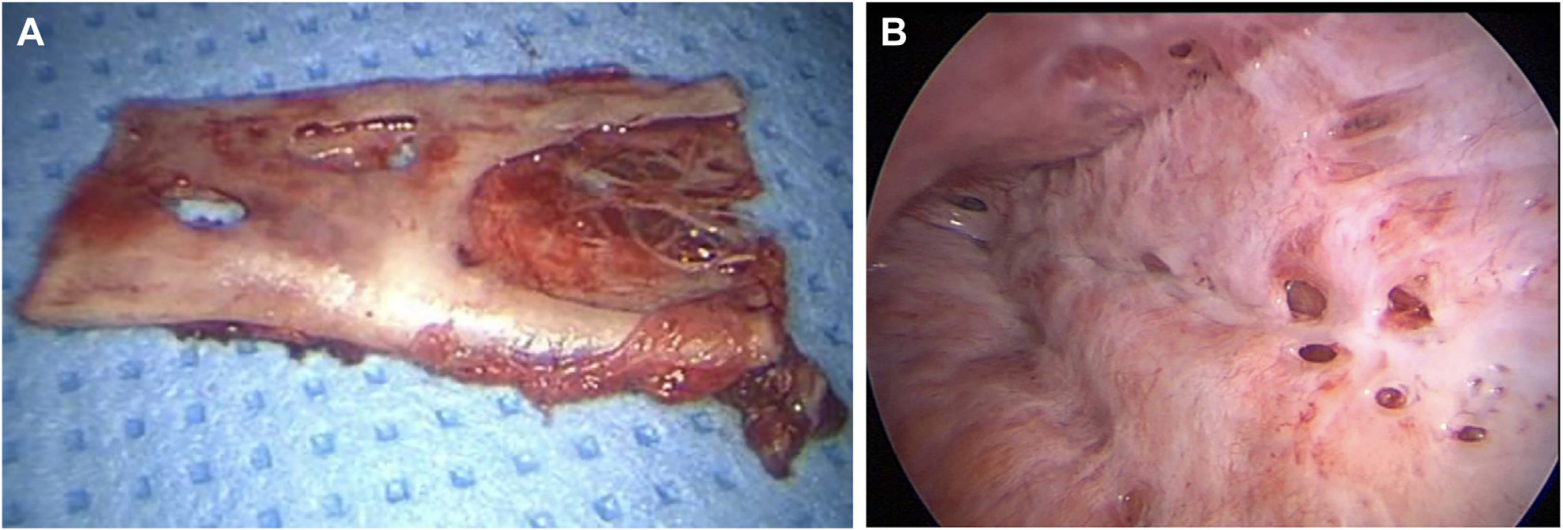
(A) Endoscopic image of excision biopsy taken from posterior aspect of left rib. (B) Endoscopic image of left thoracic wall showing multiple small holes communicating with left flank.

Unfortunately, despite these numerous interventions, there was progressive recollection of the left sided chylothorax and so the patient underwent left thoracotomy, rib excision, decortication, pleurectomy, and talc pleurodesis. Intraoperatively, the left flank was explored and found to harbor a large smooth lined cavity that was communicating with the left pleural cavity via multiple small holes (Figure 3B). The rib was almost totally destroyed. The holes were closed via the use of nonabsorbable sutures and glue.

This final procedure successfully resolved the chylothorax and no further pleural recollection was identified at follow-up. Ongoing management needs for this patient’s GSD related to persistent bone pains for which she was commenced on bisphosphonate infusions a year later. Five years after the last surgery, thoracic imaging of our patient showed stable changes with no evidence of intrathoracic recollection.

## Comment

GSD is a rare disorder that can present with a number of clinical manifestations. Because of its obscurity, diagnosis is challenging and, as such, warrants a high grade of clinical suspicion and diagnostic examination. The presence of a chylothorax alongside distinctive bone lesions is suggestive of the disease. However, a bone biopsy is often still required in order to confirm the diagnosis.^3^ The patient in our case was initially managed as a spontaneous chylothorax but subsequent diagnosis of GSD was made based upon revelation of concomitant features and significant radiologic findings; ultimate confirmation came from the bone biopsy.

Patients can present with a wide variety of symptoms depending on the affected bone.^4^ Chylothorax as an initial presentation has been reported, and its disease course can even prove fatal in some cases.^5^ Bone fractures are not uncommon as another complication of GSD. This patient’s initial presentation was increasing shortness of breath secondary to pleural effusion, the fluid of which turned out to be chylous. This was all on a background of previous spinal fractures for which she had undergone spinal fixation surgery.

Management of patients with chylothorax secondary to GSD can be different from other causes of chylothorax. Whereas thoracic duct ligation is a valid treatment of patients with idiopathic chylothorax,^6^ it does not seem to be as effective in the management of chylothorax secondary to GSD since the lymph is originating from the bone and not the usual lymphatic channels. In this particular case, different treatment modalities were used including surgery and radiotherapy. Targeting the affected bone with radiotherapy for the treatment of GSD has been previously reported and, indeed, when combined with surgical intervention, radiotherapy can be seen to slow the progress of the disease.^7^ Because GSD involves the progressive destruction and resorption of bone, patients can present with severe bone pains. Studies have found that such patients may benefit from bisphosphonate administration in order to alleviate these pains^8^; it was and continues to be used for our patient and has proven effective in controlling the symptoms of GSD.

In conclusion, GSD is a rare disease that can develop a number of severe complications including a chylothorax. Management via surgical and radiotherapeutic techniques can provide long-term remission, however, other modalities may be needed in order to optimize analgesic requirements.
